# Oral and Topical Anti-Inflammatory and Antipyretic Potentialities of *Araucaria bidiwillii* Shoot Essential Oil and Its Nanoemulsion in Relation to Chemical Composition

**DOI:** 10.3390/molecules26195833

**Published:** 2021-09-26

**Authors:** Mohamed F. Abdelhameed, Gihan F. Asaad, Tamer I. M. Ragab, Rania F. Ahmed, Abd El-Nasser G. El Gendy, Sahar S. Abd El-Rahman, Abdelbaset M. Elgamal, Abdelsamed I. Elshamy

**Affiliations:** 1 Pharmacology Department, National Research Centre, Dokki, Giza 12622, Egypt; mf.abdelhameed@nrc.sci.eg (M.F.A.); gf.asaad@nrc.sci.eg (G.F.A.); 2 Chemistry of Natural and Micro and al Products Department, National Research Centre, Dokki, Giza 12622, Egypt; tamerragab2006@gmail.com; 3 Chemistry of Natural Compounds Department, National Research Center, 33 El Bohouth St., Dokki, Giza 12622, Egypt; rf.ali@nrc.sci.eg; 4 Medicinal and Aromatic Plants Research Department, National Research Centre, 33 El Bohouth St., Dokki, Giza 12622, Egypt; aggundy_5@yahoo.com; 5 Department of Pathology, Faculty of Veterinary Medicine, Cairo University, Giza 12211, Egypt; saharsamirmah@cu.edu.eg; 6 Department of Chemistry of Microbial and Natural Products, National Research Centre, 33 El-Bohouth St., Dokki, Giza 12622, Egypt

**Keywords:** anti-inflammatory, *Araucaria bidiwillii*, essential oil, nanoemulsion, inflammatory cytokines, immunohistochemical, antipyretic

## Abstract

Different parts of *Araucaria bidiwillii* (bunya pin) trees, such as nuts, seeds, bark, and shoots, are widely used in cooking, tea, and traditional medicines around the world. The shoots essential oil (EO) has not yet been studied. Herein, the chemical profile of *A. bidiwillii* shoots EO (ABSEO) was created by GC–MS analysis. Additionally, the in vivo oral and topical anti-inflammatory effect against carrageenan-induced models, as well as antipyretic potentiality of ABSEO and its nanoemulsion were evaluated. Forty-three terpenoid components were identified and categorized as mono- (42.94%), sesqui- (31.66%), and diterpenes (23.74%). The main compounds of the ABSEO were beyerene (20.81%), α-pinene (16.21%), D-limonene (14.22%), germacrene D (6.69%), β-humulene (4.14%), and sabinene (4.12%). The ABSEO and its nanoemulsion exhibited significant inflammation suppression in carrageenan-induced rat paw edema model, in both oral (50 and 100 mg/kg) and topical (5% in soyabean oil) routes, compared to the control and reference drugs groups. All the results demonstrated the significant inflammation reduction via the inflammatory cytokines (IL-1β and IL8), nitrosative (NO), and prostaglandin E2 (PGE2) supported by the histopathological studies and immunohistochemical assessment of MMP-9 and NF-κβ levels in paw tissues. Moreover, the oral administration of ABSEO and its nanoemulsion (50 and 100 mg/kg) exhibited antipyretic activity in rats, demonstrated by the inhibition of hyperthermia induced by intramuscular injection of brewer’s yeast. These findings advised that the use of ABSEO and its nanoemulsion against numerous inflammatory and hyperthermia ailments that could be attributed to its active constituents.

## 1. Introduction

Nature products have represented the main resources of the backbone of human needs for life from the beginning of civilization, including foods and remedies [1]. Around 80% of humanity has used ≈21,000 medicinal herbs for the treatment of diseases [2]. Essential oils (EOs) have played an important role in the treatment of several diseases [3]. EOs represented one of the main targets of the researchers in the fighting of the different diseases especially inflammations and tumors [2].

Inflammation is a physiological response of the body’s immune system against several insults, including pathogens, toxins, destroyed cells, and/or radiation [4]. This reaction helps the body to diminish harm and aid in the repair of tissue injury. Moreover, the inflammatory reaction is characterized by many cellular and molecular cascades [5]. Cellular inflammation represents the principal process in various diseases, leading to inappropriate cell death, organ-specific damage, or the development of different cancers. Chronic inflammation is implicated in the progress of several disorders, such as metabolic, cardiac and digestive system and autoimmune disorders, as well as Alzheimer’s disease [6,7].

Acute inflammation is as well attributed to the liberation of reactive oxygen species (ROS) [8]. Additionally, previous research has reported the implication of several cytokines in promoting inflammation as interleukin-1 β (IL-1β) and tumor necrosis factor-α (TNF-α) [6,9]. Nuclear factor-kappa B (NF-κB) has a vital role in the maintenance and regulation of acute inflammation. A gene’s expression of many proinflammatory cytokines is mediated by NF- κB [10].

It was reported that Cyclooxygenase-2 (COX-2) is a critical enzyme that controls the production of inflammatory prostaglandins [6]. Furthermore, much evidence had been recorded that heme oxygenase-1 (HO-1) plays a crucial role in the anti-inflammatory mechanisms of cells [11] by protecting cells and tissues from oxidative stress damage [12]. The enhancement of nuclear factor erythroid 2-related factor 2 (Nrf2) could prevent the worsening of inflammation [13] and potentiate the anti-inflammatory effect [14].

Many biological activities were recorded for the different extracts of *Araucaria* plants (family: Araucariaceae), such as antiviral, anti-inflammatory, antiulcerative, neuro-protective, antidepressant, and antimicrobial activities [15,16]. Chemically, many chemical constituents were isolated and characterized from *Araucaria* species, such as terpenes, especially sesqui- and di-types, and phenolic compounds comprising biflavonoids, isoflavonoids, and phenylpropanoids [17,18]. Terpene-enriched EOs were reported as one of the main components of *Araucaria* species with several biological potentialities, such as anticancer, anti-inflammatory, antipyretic, antiproliferation, and antibacterial [1,19,20,21]. 

Nuts, seeds, bark, and shoots of *A. bidiwillii* (bunya pin) trees were widely used in food and tea around the world, especially in Australia and South Africa [22]. In folk medicine around the world, the bark is used for amenorrhea treatment as well as body and/or steam wash. The EOs of Egyptian foliage and Australian leaf *A. bidiwillii* were earlier documented by [1,19]. Both research teams found that these EOs involve diterpenes as the main components. Recently, El-Hawary et al. [23] described that the methanolic extract of *A. bidiwillii* is very rich in flavonoids and phenolic acids, with significant antioxidant activity.

Recently, the enhancement of the plant extracts, EOs, and isolated metabolites has taken the attention of scientists via preparation of nanoforms, such as nanoextracts, nanoemulsions, and encapsulation [6]. The most significant improvements in the extracts in drug delivery were documented by varying the nanoparticles size, ranging from 10 to 1000 nm [6]. These biological improvements included bioavailability, residence time, the cognate receptors clearance, targeting, safety, drug dose, and side effects [6,20].

The targets of the current work were established based upon the significant antioxidant and cytotoxic activity of the EO and extracts of different parts of *A. bidiwillii* [1], and the potent anti-inflammatory and antipyretic potentialities of EO of *A. heterophylla* [20,24]. Additionally, the previous documents indicated the potential role of the nanoforms of the different plant extracts in the modification of the biological potentialities of these extracts [20,24]. Thus, the main outlines of current work were (i) identification of the chemical composition of the tree shoots, *A. bidiwillii*, (ABSEO) cultivated in Egypt, (ii) formulation of the extracted of EO in nanoemulsion form, (iii) evaluation of the oral and topical anti-inflammatory as well as antipyretic potentialities of EO and its nanoemulsion depending upon biochemical, pathological and immunohistochemical analysis

## 2. Materials and Methods

### 2.1. Plant Materials

The shoots of the cultivated tree, *Araucaria bidiwillii* (family Araucariaceae) were collected in October 2019 from El-Orman Botanic garden, Giza, Egypt (30°01′45″ N 31°12′47″ E). The collection of the shoots was carried out by Prof. Dr. Mohamed A. Gibali, a senior botanist, El-Orman Botanic Garden, Giza, Egypt. A plant specimen (x00917-AUB1006-ABSEOB) was deposited in the El-Orman Botanic Garden Herbarium.

### 2.2. Drugs and Chemicals

Carrageenan, paracetamol, and diclofenac were purchased from Sigma Aldrich (Merck, Darmstadt, Germany). Brewer’s yeast was purchased from AngelYeast Co., Ltd. (Yichang, Hubei, China). ELISA kits were used for the assessment of interleukin-1β and interleukin-8 (IL-1β and IL-8; Cloud-Clone, Katy, TX 77494, USA). Nitric oxide (NO) was determined spectrophotometrically using chemical methods. All chemicals used were of the highest commercial grade available.

### 2.3. Essential Oil Extraction, GC–MS Analysis and Chemical Constituents’ Identification

The fresh shoots of *A. bidiwillii* (200 g) were subjected to hydro-distillation via a Clevenger apparatus. After three hours, the oil was separated via *n*-hexane and then dried by 0.5 g anhydrous sodium sulphate (Na_2_SO_4_). Three ABSEO samples were obtained via repeating this procedure for three different plant samples and then saved in glass vials in the refrigerator at 4 °C for analysis.

The chemical profile of the three extracted ABSEO separately was established based upon the gas chromatography/mass spectrometry (GC–MS) according to the earlier documented protocol [25].

### 2.4. ABSEO Nanoemulsion Preparation

Tween 80 was used as a non-ionic surfactant to prepare a nanoemulsion of ABSEO *A. bidiwillii*. As the organic phase, Tween 80 was added to EO (1:1 *w*/*w*). At 25 °C, the organic phase mixture was steadily applied in droplets to distilled water (aqueous phase), stirring vigorously. Ultrasonic (Sonics & Materials, Inc., 53 Church Hill Rd., Newtown, CT, USA) with a probe diameter of 13 mm was used to sonicate the prepared emulsion at 20 °C for 15 min at a high frequency of 20kHz and a power output of 750 W [26].

### 2.5. Droplet Size and Zeta Potential Analysis

At 23 °C, a dynamic light scattering (DLS) instrument (PSS, Santa Barbara, CA, USA) was used to calculate the average size, size distribution, and zeta potential using the 632.8 nm line of a HeNe laser as the incident light with an angle of 90° and the zeta potential with an external angle of 18.9° [27].

### 2.6. Biological Experimental Section

#### 2.6.1. Experimental Animals and Ethical Statements

The adult male Wistar rats and mice were purchased from the animal house, National Research Centre, Egypt. The housing of the rats was carried out in metal cages at the animal house, National Research Centre, Egypt under strictly standard conditions as well as the availability of water and food to rats. The study was conducted in accordance with the Declaration of Helsinki, and the protocol was approved by the National Research Center Committee, Egypt (approval no: CU/F/16/21).

#### 2.6.2. Evaluation of Acute Toxicity 

Thirty-six Swiss albino mice were divided into six groups (6 mice each), one group of each sex, Groups 1 and 2, were orally administered separately with ABSEO, while groups 3 and 4 represented both sexes administered with ABSEO nanoemulsion up to 2 g/kg step gradient doses, and Groups 5 and 6 (control of both sexes) were orally administrated by the same volumes of distilled water with 2 drops of Tween 80. The animals were observed for mortality 24 h later. Additionally, mice were observed for any changes in the skin, fur, respiratory, circulatory, or behavioral pattern [20,24].

#### 2.6.3. Anti-Inflammatory and Antipyretic Effects of Oral Administration of ABSEO and Nanoemulsion

##### Carrageenan-Induced Rat Paw Edema

Forty-two male Wistar rats (150–200 bw) were divided randomly into seven groups (*n* = 6). Group 1 was kept as normal control (1ml saline per os) and injected with 0.1 mL saline (1%) at the sub plantar region of the left hind paw; Group 2 served as positive control administered 1ml saline per os and injected with 0.1 mL carrageenan (1% *w*/*v*) at sub plantar region of the left hind paw according to the method of [28]. Group 3 was given oral administration of diclofenac (30 mg/kg) as standard drug [9]. Groups 4 and 5 were given ABSEO in two dose levels (50 and 100 mg/kg, respectively). Groups 6 and 7 were given ABSEO nanoemulsion in two dose levels (50 and 100 mg/kg, respectively). Thirty minutes later, Groups 3–7 were injected with 0.1 mL carrageenan solution (1% (*v*/*v*) in saline at the sub planter area of the left hind paw. Then, after 1, 2, 3, and 4 h of carrageenan injection, paw thickness was measured for all the rats using MNT-150 vernier caliper (Jiangsu Goldmoon Industry Co., Ltd.- Shanghai, China) and expressed in milliliters [29]. Edema rate and inhibition rate were calculated at the mentioned intervals using the following equations [30]:

Edema rate (%) = Vt − Vo/Vo and Inhibition rate (%) = Ec − Et/Ec

where Vo is the thickness before carrageenan injection (mL); Vt is the thickness at t hour after carrageenan injection (mL); Ec is the edema rate of the control group; and Et is the edema rate of the treated group.

Four hours after carrageenan injection, the rats were sacrificed by cervical dislocation under anesthesia with a high dose of ketamine from the overnight fasted animals and examined paws (hind left paws) were cut at the level of the calcaneus bone. The paws were then degloved to remove the bones and immediately frozen in liquid nitrogen and kept till needed [9].

#### 2.6.4. Antipyretic Effect

Forty-two rats were divided into seven groups (*n* = 6). Rectal temperature was recorded using a digital thermometer before yeast injection. Each animal was then injected intramuscularly with a pyrogenic dose of Brewer’s yeast (1 mL/100 g bwt of 44% yeast suspension in saline) [31]. The temperature was then measured 24 h following the yeast injection, and this was considered the baseline of elevated body temperature, to which the antipyretic effect will be compared. Rats that expressed a >0.3 °C increase in rectal temperature were considered pyretic and selected to complete the experiment. A single oral administration of the two doses of the ABSEO (50 and 100 mg/kg, respectively), its nanoemulsion (50 and 100 mg/kg, respectively), paracetamol (150 mg/kg) [32], and diclofenac (30 mg/kg) and saline (positive control) were carried out, and the rectal temperature was determined after 30, 60, and 120 min of intervention [33].

#### 2.6.5. Topical Anti-Inflammatory Effect of ABSEO and Nanoemulsion

##### Carrageenan-Induced Rat Paw Edema

Thirty male Wistar rats (150–200 g) were divided randomly into five groups (*n* = 6). Group 1 was kept as normal control (soyabean oil topically). Group 2 served as a positive control and was given soyabean oil topically injected with 0.1 mL carrageenan (1% *w*/*v*) at the sub plantar region of the left hind paw [28]. For Group 3, diclofenac 5% in soyabean oil was applied topically. Groups 4 and 5 received 5% ABSEO in soyabean oil and 5% ABSEO nanoemulsion in soyabean oil, respectively, via topical application. Thirty minutes later, Groups 3–5 were injected with carrageenan as the same method mentioned above. After 1, 2, 3, and 4 h of carrageenan injection, paw thickness [29], edema rate, and inhibition [30] were measured and calculated as described above. Four hours after 1% carrageenan injection, rats were sacrificed as mentioned above. The paws were then degloved to remove the bones and immediately frozen in liquid nitrogen and kept till needed [9].

#### 2.6.6. Homogenate Preparation

The tissues were washed thoroughly and rinsed with ice. They were gently blotted between the folds of a filter paper and weighed in an analytical balance. Homogenate (10%) was prepared in 0.05 M phosphate buffer (pH 7) using a polytron homogenizer at 40 °C. The homogenate was centrifuged at 10,000 rpm for 20 min for removing the cell debris, unbroken cells, nuclei, erythrocytes, and mitochondria. Protein content in the tissue was determined according to the method of [34], using Genei, Bangalore, protein estimation kit. The supernatant (cytoplasmic extract) was used for the estimation of IL-1β and IL-8 protein levels according to manual instructions. All ELISA kits were measured by ELISA reader; color absorbance was read at OD range 450 to 630 nm using an enzyme-linked immuno-sorbent assay (ELISA) plate reader (Stat Fax 2200, Awareness Technologies, Palm City, FL, USA). Nitric oxide (NO) was determined spectrophotometrically using chemical methods. All chemicals used were of the highest commercial grade available.

#### 2.6.7. Assessment of Inflammatory Cytokines and Nitrosative Biomarker 

IL-1β and IL-8 were determined using specific ELISA kits according to the manufacturer’s instructions. All the parameters are measured using OD 450 nm. NO content was determined according to [35] via the assessment of total nitrite levels; the only stable end product of the autoxidation of NO, vanadium (III), reduces nitrate to nitrite and/or nitric oxide, and both are captured by Griess reagents (premixed 50 μL sulfanilamide (2% in 5% HCl) and 50 μLN-(1-Naphthyl) ethylenediamine dihydrochloride (0.1%)). The Griess reaction forms a chromophore, which results in a measurable pink metabolite measured spectrophotometrically at 540 nm.

#### 2.6.8. PGE2 Gene Expression Analysis

Tissues from all different groups were homogenized and total RNA was extracted with Direct-zol RNA Miniprep Plus (Cat# R2072, ZYMO RESEARCH CORP., Irvine, CA 92614, USA) and then quantity and quality were assessed by Beckman dual spectrophotometer (Beckman, Watertown, MS, USA).

SuperScript IV One-Step RT-PCR kit (Cat# 12594100, Thermo Fisher Scientific, Waltham, MA, USA) was utilized for reverse transcription of extracted RNA followed by PCR. A 48-well plate StepOne instrument (Applied Biosystem, Foster, CA, USA) was used in a thermal profile as follows: 10 min at 45 °C for reverse transcription, 2 min at 98 °C for RT inactivation and initial denaturation by 40 cycles of 10 s at 98 °C, and 10 s at 55 °C and 30 s at 72 °C for the amplification step. After the RT-PCR run, the data were expressed in cycle threshold (Ct) for the target genes and housekeeping gene. Normalization for variation in the expression of each target gene, p21 and p16, was performed referring to the mean critical threshold (CT) expression value of β-actin housekeeping gene by the mean critical threshold (ΔΔCt) method. The relative quantitation (RQ) of each target gene is quantified according to the calculation of the 2-∆∆Ct method. 

#### 2.6.9. Hematoxylin and Eosin (H&E) Staining and Inflammation Scoring 

Soft tissues of the paw of all groups were excised and fixed in 10% neutral buffer formalin. Formalin-fixed specimens were routinely processed for obtaining paraffin blocks. The specimens were dehydrated in graded concentrations of ethanol, cleared in xylene, embedded in paraffin, sectioned at 3–4 mm thickness, and finally stained with Hematoxylin and Eosin (H&E) stain according to [36]. The stained sections were examined blindly by light microscope, and the degree of paw damage, as well as the degree of inflammatory reaction, was evaluated and scored according to [37] with a score from 0 to 5, where: 0 = no inflammation, 1 = mild inflammation, 2 = mild/moderate inflammation, 3 = moderate inflammation, 4 = moderate/severe inflammation, and 5 = severe inflammation.

#### 2.6.10. Immunohistochemical Examination

Immunohistochemistry for MMP-9 and NF-κB p65 expression was formed using the avidin-biotin-peroxidase technique. Paraffin sections were de-waxed and dehydrated, followed by heat treatment for antigen retrieval. Sections were incubated with monoclonal antibodies of MMP-9 and NF-κB p65 (Dako Corp, Carpenteria, Santa Clara, CA 95051, USA) at dilutions of (1:200 and 1:100, respectively) as well as the other reagents adopted for the avidin-biotin-peroxidase technique (Vactastain ABC peroxidase kit, Vector Laboratories, Burlingame, CA 94010, USA) according to methods mentioned by [38]. Visualization of each marker expression was achieved by Chromagen 3,3 -diaminobenzidine tetrahydrochloride (DAB, Sigma Chemical Co., Merck, Darmstadt, Germany). Image analysis software (Image J, 1.46a, NIH, USA) was used for quantitative analysis of the immune stained sections by measuring the optical density of the positive brown color in 5 microscopic fields (the area for each microscopic field is 18.893 Sqmm).

#### 2.6.11. Statistical Method

All results of biology were expressed as mean ± standard error of the mean (SEM). Statistical analysis was performed by one-way analysis of variance (ANOVA) followed by a Tukey test for confirmation and multiple comparisons. 𝑃 < 0.05 was assumed to denote statistical significance. The statistical analysis was performed using Graph-Pad Prism software (Version 7.00).

## 3. Results

### 3.1. Chemical Profile of ABSEO 

**Figure 1:**
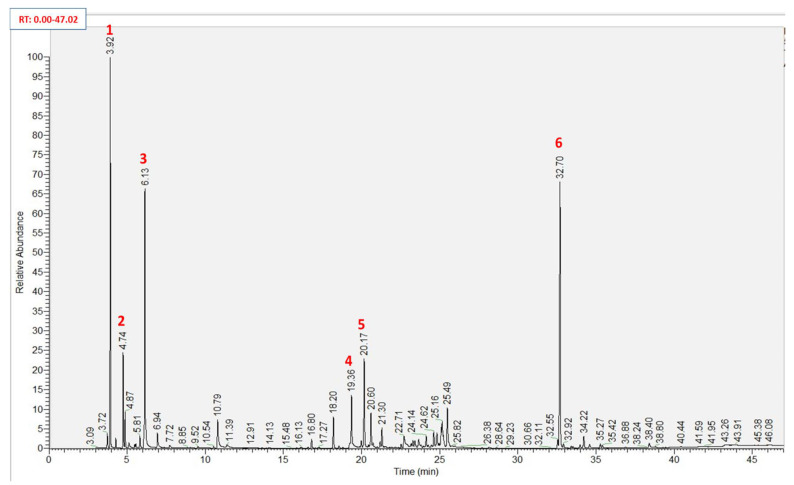
GC–MS chromatogram of ABSEO of shoots of *Araucaria bidiwillii* cultivated in Egypt. Main components were numbered (**1**–**6**); α-Pinene (**1**), Sabinene (**2**), d-Limonene (**3**), β-Humulene (**4**), Germacrene D (**5**), and Beyerene (**6**).

The hydrodistillation of the fresh shoots of *A. bidiwillii* cultivated in Egypt afforded ≈0.47% (*v*/*w*) of a dark yellow oil with a pungent odor. Both qualitative and quantitative analysis of the extracted ABSEO based upon GC–MS revealed to the identification of forty-three components counted 98.34% of the overall EO mass (Figure 1). All the identified compounds were listed in Table 1 along with their retention times (Rt.), relative concentrations (Rel. Conc. %), molecular formula (M.F.), and kovat’s indexes (KI.) in addition to the relative concentrations of the combined constituents in the previous studies of *A. bidiwillii*’s different parts.

### 3.2. Essential Oil Nanoemulsion

**Figure 2:**
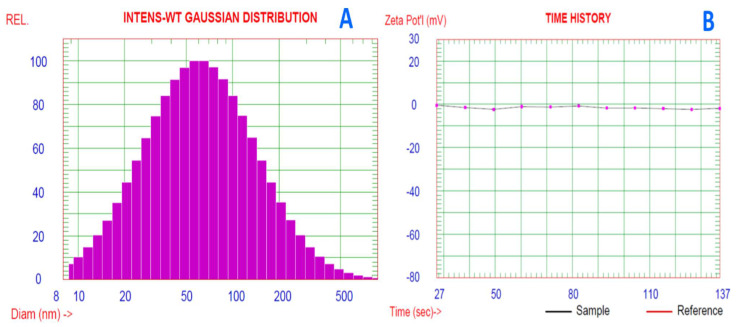
(**A**) Particle size distribution and (**B**) zeta analysis of *Araucaria bidiwillii* EO nanoemulsion (mean diameter = 106 nm).

Nanoemulsion of ABSEO was prepared by deionized water and a hydrophilic surfactant polysorbate 80 (Tween 80). The prepared nanoemulsion’s droplet size distribution was calculated. The mean droplet size diameter was 86.9 ± 0.846 nm, with 25% of the distribution less than 35.4 nm, 50% < 61.7 nm, 75% < 108.4 nm, 80% < 124.7 nm, while 90% of the distribution was less than 180.3 nm (Figure 2A). The zeta potential average value of the nanoemulsion was −1.84 mV (Figure 2B).

### 3.3. Results of Oral Anti-Inflammatory Effect of ABSEO and Its Anoemulsion

The results showed that group-administered ABSEO (50 mg/kg) exerted very week edema inhibition percentages (10.24%) after 3 h interval of carrageenan injection. The ABSEO administered at 100 mg/kg showed a better effect only after 1 h of carrageenan injection (25.04%) than those administered 50 mg/kg, and then this effect was declined till the end of the experiment. The groups administered ABSEO nanoemulsion (50 and 100 mg/kg) showed a gradual increase in % of edema inhibition till 4 h of carrageenan injection. After 1 h, all drugs except ABSEO (50 mg/kg) showed better EI% in the range 17.8–25.6% compared with diclofenac 13.36%. Meanwhile, after a 4 h interval, the EI% of ABSEO nanoemulsion (50 and 100 mg/kg) was increased to reach the range of 47.5–58.2% in comparison to diclofenac, whose EI% was 76.7% (Table 2).

#### 3.3.1. Effect on Interleukin 1β (IL-1β) and 8 (IL-8)

**Figure 3:**
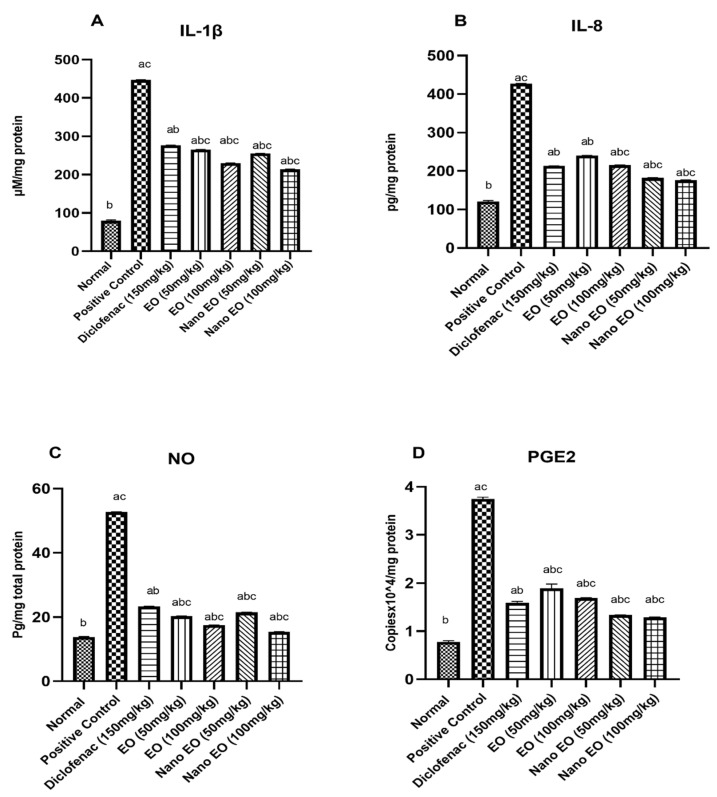
Effect of oral administration of ABSEO and ABSEO nanoemulsion (50–100 mg/kg) on (**A**) IL-1β, (**B**) IL-8, (**C**) Nitric oxide (NO), and (**D**) PGE2 RNA expression induced by subplantar injection of carrageenan in the hind left paw of rats as compared to standard drug; diclofenac. Data are expressed as (mean ± SE). Statistical analysis was performed by one-way ANOVA and confirmed by Tukey test. *p* < 0.05 was assumed to denote statistical significance. a—Significantly different from normal group at *p* < 0.05. b—Significantly different from positive control group at *p* < 0.05. c—Significantly different from diclofenac group at *p* < 0.05.

The positive control group injected with 0.1 mL carrageenan (1% *w*/*v*) significantly (*p* ˂ 0.05) increased IL-1β (447 ± 0.71 pg/mg protein) and IL-8 (427 ± 3.72 pg/mg protein) as compared to normal control group (79.6 ± 2.16 pg/mg protein and 120.6 ± 3.12 pg/mg protein, respectively). Oral treatment with diclofenac (Group 3) at a dose of 30 mg/kg significantly (*p* ˂ 0.05) decreased the induced IL-1β (276 ± 2.7 pg/mL protein) and IL-8 (213 ± 1.7 pg/mL protein). Oral treatment (Groups 4–7) with ABSEO (50–100 mg/kg) and ABSEO nanoemulsion (50–100 mg/kg), respectively, significantly decreased the elevated IL-1β (265 ± 4.21, 230 ± 2.75, 255 ± 2.75, and 214 ± 2.35 pg/mg protein, respectively (Figure 3A)) as well as IL-8 (240 ± 1.78, 215 ± 2.01, 182 ± 3.84, and 176.6 ± 2.55 pg/mg protein, respectively (Figure 3B)), as compared to the positive control group.

#### 3.3.2. Effect of Nitric Oxide (NO)

The positive control group injected with 0.1 mL carrageenan (1% *w/v*) significantly (*p* ˂ 0.05) increased NO (52.7 ± 5.02 μM/mg protein) as compared to the normal control group (13.8 ± 0.15 μM/mg protein). Oral treatment with standard diclofenac (Group 3) at a dose of 30 mg/kg significantly (*p* ˂ 0.05) decreased the induced NO (23.4 ± 1.7 pg/mL) when compared to the positive control group. Oral treatment (Groups 4–7) with ABSEO (50–100 mg/kg) and ABSEO nanoemulsion (50–100 mg/kg), respectively, significantly decreased the elevated NO (20.32 ± 0.58, 17.5 ± 1.01, 21.5 ± 1.9, and 15.4 ± 0.55 μM/mg protein, respectively) as compared to the positive control group (Figure 3C**)**. 

#### 3.3.3. Quantification of Prostaglandin E2 (PGE2) RNA Expression

Prostaglandin E2 (PGE2) RNA expression was assessed in the tissue homogenate. Our results showed a significant increase (*p* < 0.05 ) in PGE2 RNA expression in the positive control group (3.74 ± 0.04 Copiesx10^4/mg total protein) when compared to the normal control group (0.78 ± 0.02 Copiesx10^4/mg total protein), showing an increase in PGE2 RNA expression to 353.14% in the positive control group as compared to the normal control group. Oral administration of diclofenac (30mg/kg) showed a significant reduction (*p* < 0.05) in PGE2 RNA expression (1.59 ± 0.03 Copiesx10^4/mg total protein) as compared to the positive control group. Oral administration of ABSEO (50 and 100 mg/kg) and ABSEO nanoemulsion (50–100 mg/kg) showed a significant reduction (*p* < 0.05) in PGE2 RNA expression (1.89 ± 0.08, 1.69 ± 0.01, 1.33 ± 0.004, and 1.29 ± 0.008 Copiesx10^4/mg total protein, respectively), as compared to positive control group (Figure 3D). 

### 3.4. Result of Antipyretic Effect

**Figure 4:**
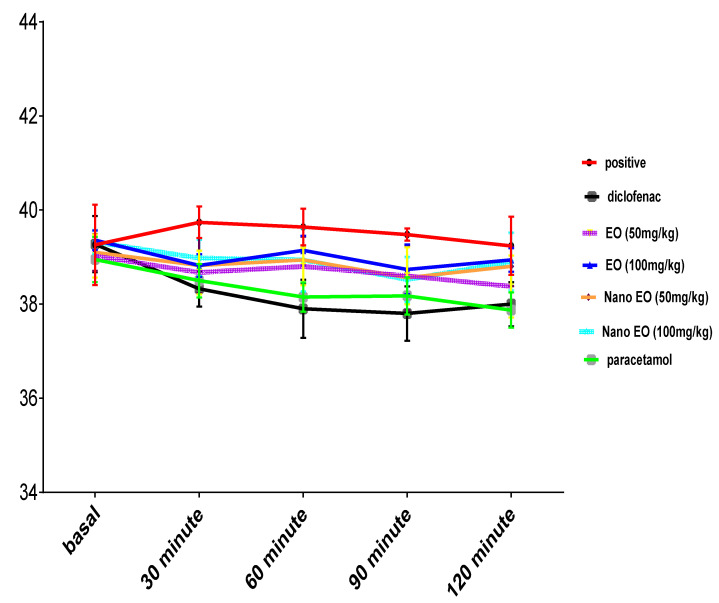
Effect of oral administration of ABSEO and ABSEO nanoemulsion (50–100 mg/kg) on pyrogenesis induced by intramuscular injection of rats with brewer’s yeast as compared to standard drug; diclofenac and paracetamol. Data are expressed as (mean ± SE). Statistical analysis was performed by one-way ANOVA and confirmed by Tukey test. *p* < 0.05 was assumed to denote statistical significance.

Intramuscular injection of brewer’s yeast suspension significantly elevated the rectal temperature after 24 h of administration. Oral administration of ABSEO and its nanoemulsion at both doses (50 and 100 mg/kg) showed a gradual decrease in rectal temperature induced by the intramuscular injection of brewer’s yeast injection until 90 min after, while the administration of standard drugs diclofenac (30 mg/kg) and paracetamol (150 mg/kg) continued to reduce fever until the last measurement (120 min). By comparing all the groups with the positive control group, we found that all the drugs given showed a significant (*p* ˂ 0.05) reduction in the temperature (Figure 4).

### 3.5. Results of Topical Anti-Inflammatory Effect of ABSEO and Its Nanoemulsion 

#### 3.5.1. Carrageenan-Induced Rat Paw Edema

The efficacy of the ABSEO and its nanoemulsion was expressed as the % edema inhibition (EI %) consequently after 1, 2, 3, and 4 h compared to standard diclofenac. The effect of topical application of diclofenac appeared only after 1 h following carrageenan injection with percent of inhibition of 27.09%, and in the same time interval, the drugs (ABSEO and its nanoemulsion) applied topically showed a better effect than that exerted by diclofenac, showing a percent of inhibition of 32.47 and 51.02%, respectively. The anti-inflammatory effect of ABSEO topical application persisted till the fourth hour following carrageenan injection, showing a percent of inhibition of 59.87%. On the other hand, ABSEO nanoemulsion showed inhibition at the first hour of 51.02%, but the anti-inflammatory effect was declined till the fourth hour following carrageenan injection (Table 3).

#### 3.5.2. Effect on Interleukin 1β (IL-1β), and 8 (IL-8) 

**Figure 5:**
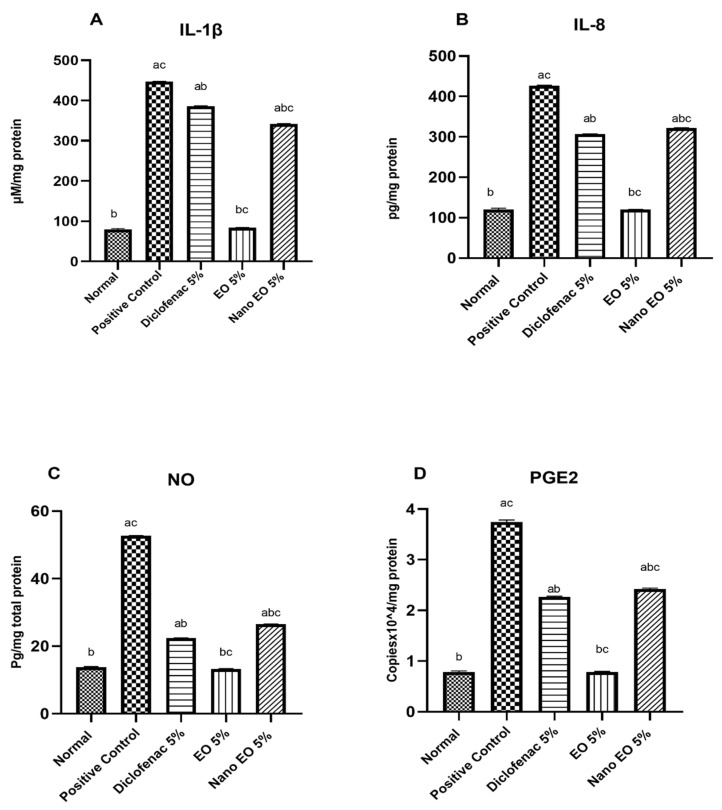
Effect of topical application of ABSEO (5%) and nanoemulsion (5%) on (**A**) IL-1β, (**B**) IL-8, (**C**) Nitric oxide (NO), and (**D**) PGE2 RNA expression, induced by intraplantar injection of carrageenan in the hind left paw of rats as compared to standard drug diclofenac. Data are expressed as (mean ± SE). Statistical analysis was performed by one-way ANOVA and confirmed by Tukey test. *p* < 0.05 was assumed to denote statistical significance. a—Significantly different from normal group at *p* < 0.05. b—Significantly different from positive control group at *p* < 0.05. c—Significantly different from diclofenac group at *p* < 0.05.

The positive control group injected with 0.1 mL carrageenan (1% *w/v*) showed significantly (*p* ˂ 0.05) increased IL-1β (437 ± 0.51 pg/mg protein) and IL-8 (411 ± 2.92 pg/mg protein) as compared to the normal control group (81.6 ± 4.16 pg/mg protein and 118.36 ± 5.12 pg/mg protein, respectively). Topical application of standard diclofenac 5% (Group 3) significantly (*p* ˂ 0.05) decreased the induced IL-1β (386 ± 0.51 pg/mL) and IL-8 (307 ± 12.7 pg/mL) when compared to the positive control group. Topical application of ABSEO and its nanoemulsion (5% in soyabean oil) (Groups 4 and 5) significantly decreased both the elevated IL-1β (84.6 ± 2.35 and 342 ± 1.21 pg/mg protein, respectively (Figure 5A)) and the elevated IL-8 (120 ± 8.7 and 322 ± 15.68 pg/mg protein, respectively (Figure 5B)) as compared to the positive control group. Our results showed that both ABSEO and its nanoemulsion showed a significant (*p* ˂ 0.05) anti-inflammatory effect as compared to standard diclofenac. In addition, it was obvious that ABSEO restored the IL-1β and IL-8 levels to normal levels (Figure 5A,B). 

#### 3.5.3. Effect of Nitrosative Biomarker (Nitric Oxide; NO)

The positive control group injected with 0.1 mL carrageenan (1% *w/v*) showed significantly (*p* ˂ 0.05) increased NO (49.5 ± 4.12 μM/mg protein) as compared to the normal control group (15.6 ± 2.15 μM/mg protein). Topical application with standard diclofenac 5% (Group 3) significantly (*p* ˂ 0.05) decreased the induced NO (22.4 ± 0.87 pg/mL) when compared to the positive control group. Topical application (Groups 4–5) with ABSEO (5%) and ABSEO nanoemulsion (5%), respectively, significantly decreased the elevated NO (13.3 ± 1.54 and 26.5 ± 1.78 μM/mg protein, respectively) as compared to the positive control group. Our results showed that ABSEO showed a significant (*p* ˂ 0.05) reduction in NO level as compared to standard diclofenac. In addition, it was obvious that ABSEO restored NO to normal levels (Figure 5C). 

#### 3.5.4. Quantification of Prostaglandin E2 (PGE2) RNA Expression

Prostaglandin E2 (PGE2) RNA expression was assessed in the tissue homogenate. Our results showed a significant increase (*p* < 0.05 ) in PGE2 RNA expression in the positive control group (3.78 ± 0.12 Copies x 10^4/mg total protein) when compared to the normal control group (0.82 ± 0.01 Copies x 10^4/mg total protein), showing an increase in PGE2 RNA expression to 460.97% in the positive control group as compared to the normal control group. Topical application of diclofenac 5% showed a significant reduction (*p* < 0.05) in PGE2 RNA expression (2.27 ± 0.01 Copies x 10^4/mg total protein) as compared to the positive control group. Topical application of ABSEO (5%) and ABSEO nanoemulsion (5%) showed a significant reduction (*p* < 0.05) in PGE2 RNA expression (0.79 ± 0.013 and 2.43 ± 0.02 Copies x 10^4/mg total protein, respectively), as compared to the positive control group. Our results showed that ABSEO showed a significant (*p* ˂ 0.05) reduction in PGE RNA expression as compared to standard diclofenac. In addition, it was obvious that ABSEO restored PGE expression to normal levels (Figure 5D).

### 3.6. Histopathological Findings

**Figure 6:**
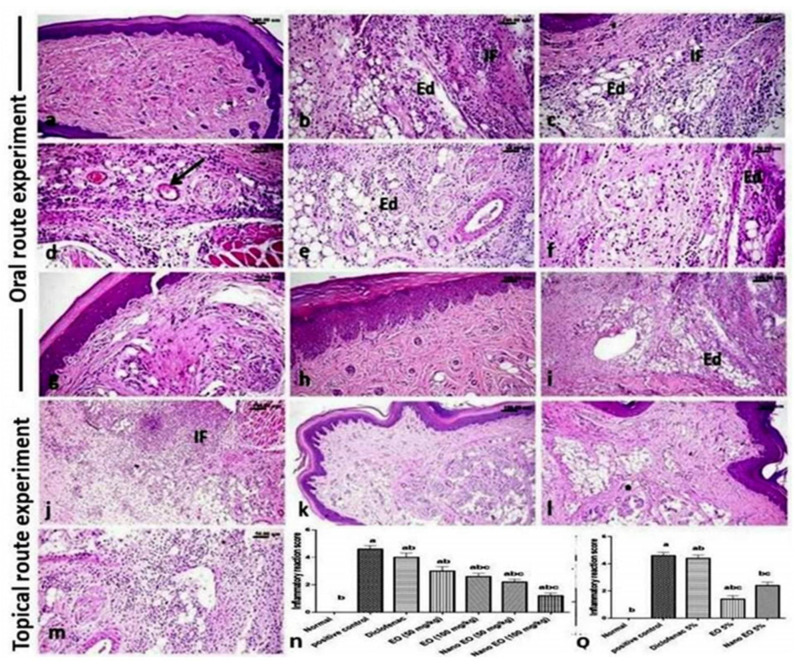
Photomicrograph of H&E-stained sections of hind paw of rats. (**a**) Control rat showing normal histological structure. (**b**–**d**) Carrageenan model rat showing; marked edema (Ed) and severe diffuse inflammatory cell infiltration (IF) admixed with many neutrophils, (**d**) vacuities with early thrombus formation (arrow). (**e**,**f**) Oral ABSEO treated carrageenan model rats at low dose (50 mg/kg) (**e**) and high dose (100 mg/kg) (**f**) showing moderate degree of decreased inflammatory reaction with still presence of edema (Ed) and infiltrated inflammatory cells (IF). (**g**,**h**) Oral ABSEO nanoemulsion treated carrageenan model rats at low dose (50 mg/kg) (**g**) and high dose (100 mg/kg), (**h**) showing a dose-related good degree of decreased to scares inflammatory reaction. (**i**,**j**) Orally (**i**) and topically (**j**) diclofenac treated carrageenan model rats showing edema and severe inflammatory reaction (IF), particularly when topically applied. (**k**,**l**) Topically applied ABSEO (5%) (**k**) and ABSEO nanoemulsion (5%) (**l**) to carrageenan model rats showing good degree of improvement of paw inflammation, particularly for the ABSEO (5%) application. (**m**) Topically applied soyabean showing marked inflammatory reaction. (**n**,**o**) The scores of inflammatory reactions in different treated groups compared to carrageenan model group. Scoring data are presented as median (max–min) using Kruskal–Wallis test followed by the Mann–Whitney *U* test. *p* < 0.05 was assumed to denote statistical significance. a—Significantly different from normal group at *p* < 0.05. b—Significantly different from positive control group at *p* < 0.05. c—Significantly different from diclofenac group at *p* < 0.05.

The hind paw of control rats showed normal histological structure (Figure 6a), while carrageenan model rats showed an increase in the thickness of the sub-epithelial layer with marked edema that disperses the dermal connective tissue fibers from each other, as well as congestion and severe inflammatory reaction. The latter, characterized by diffuse edema and mononuclear inflammatory cells infiltration (Figure 6b,c), admixed with a large number of neutrophils accompanied with marked hyalinization. The blood vessels showed vacuities characterized by vascular wall edema and vacuolation, sometimes with endothelial injury and early thrombus formation (Figure 6d). The examination of the treated groups revealed that oral administration of the ABSEO nanoemulsion had a more curative effect than that observed with the orally administrated ABSEO, while the topical application of ABSEO (5%) was more effective than the topical application of the ABSEO nanoemulsion. While the administration of diclofenac either orally or topically did not show such an obvious curative effect.

The oral treatment with ABSEO at both low (50 mg/kg) (Figure 6e) and high (100 mg/kg) (Figure 6f) doses in carrageenan model rats showed a moderate degree of decreased inflammatory reaction, still with the presence of edema and inflammatory cells that diffusely infiltrated the paw sub/cut tissue; deceased inflammatory reaction was dose related. The administration of ABSEO nanoemulsion at both low (50 mg/kg) (Figure 6g) and high (100 mg/kg) (Figure 6h) doses to carrageenan-induced inflammation rats’ paw revealed a good degree of dose-related decrease in the inflammatory reaction of the paw tissue, particularly at the high dose, which showed scarce inflammatory reaction. Additionally, the oral administration of diclofenac (Figure 6i) showed nearly no obvious retraction of the inflammatory reaction.

Concerning the topical route experiment, the topical application of soyabean as a vehicle positive control showed a marked inflammatory reaction (Figure 6j). While the topical application of ABSEO (5%) to carrageenan-induced inflamed paw resulted in good degree of improvement of paw inflammation, only few inflammatory cells were observed (Figure 6k). On the other hand, topical application of the ABSEO nanoemulsion (5%) showed mild improvement of the inflammatory reaction (Figure 6l), whereas topical application of diclofenac (Figure 6m) showed no evidence of inflammation subsiding. The scoring of the inflammatory reaction in carrageenan model rats and different treated groups is presented in Figure 6n,o.

### 3.7. Immunohistochemistry Findings

**Figure 7:**
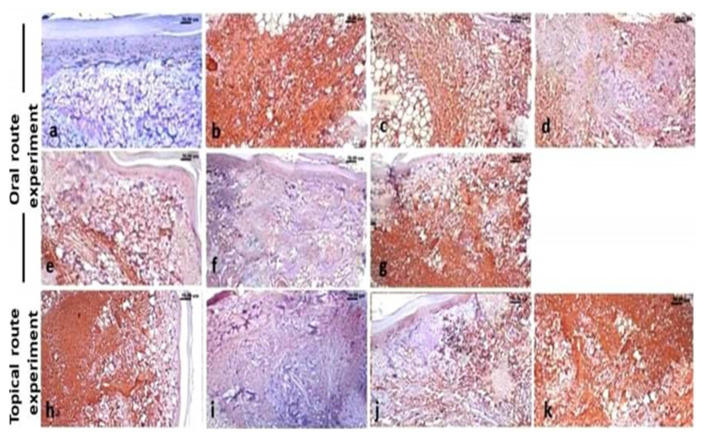
Immunohistochemical stained sections of rats’ hind paw for MMP-9 expression. (**a**) Control rat, (**b**) carrageenan model rats, (**c**,**d**) ABSEO orally treated carrageenan model rat at low (50 mg/kg) (**c**) and high (100 mg/kg) (**d**) doses, (**e**,**f**) ABSEO nanoemulsion orally treated carrageenan model rats at low (50 mg/kg) (**e**) and high (100 mg/kg) (f) doses. (**g**,**h**) Diclofenac treated carrageenan model rat orally (**g**) and topically (**h**). (**i**,**j**) Topically applied ABSEO (5%) (**i**) and ABSEO nanoemulsion (5%) (**j**) to carrageenan model rats. (**k**) Topically applied soyabean to carrageenan model rats. *p* < 0.05 was assumed to denote statistical significance.

**Figure 8:**
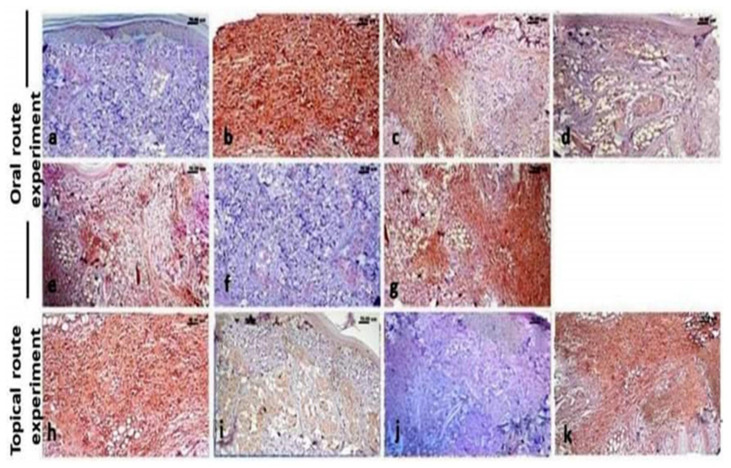
Immunohistochemical stained sections of rats’ hind paw for NF-κB p65 expression. (**a**) Control rat, (**b**) carrageenan model rats, (**c**,**d**) ABSEO orally treated carrageenan model rat at low (50 mg/kg), (**c**) and high (100 mg/kg) (**d**) doses, (**e**,**f**) ABSEO nanoemulsion orally treated carrageenan model rats at low (50 mg/kg) (**e**) and high (100 mg/kg) (**f**) doses. (**g**,**h**) diclofenac treated carrageenan model rat orally (**g**) and topically (**h**). (**i**,**j**) Topically applied ABSEO (5%) (**i**) and ABSEO nanoemulsion (5%) (**j**) carrageenan model rats. (**k**) Topically applied soyabean to carrageenan model rats. *p* < 0.05 was assumed to denote statistical significance.

The paw of normal control rats showed negative expression of both MMP-9 (Figure 7a) and NF-κB p65 (Figure 8a), while the paw tissue of carrageenan model rats showed a significantly increased expression of MMP-9 (Figure 7b) and NF-κB p65 (Figure 8b). Regarding the treated groups, the immune expression of both markers showed variable degrees of decreased expression. The oral treatment with ABSEO (revealed moderate expression of both markers particularly at the high dose (Figure 7c,d and Figure 8c,d), while oral treatment with the ABSEO nanoemulsion resulted in significant dose related decreased expression of bot markers (Figure 7e,f and Figure 8e,f), whereas the oral administration of diclofenac resulted in very mild decreased intensity of both markers’ expression (Figure 7g and Figure 8g).

**Figure 9:**
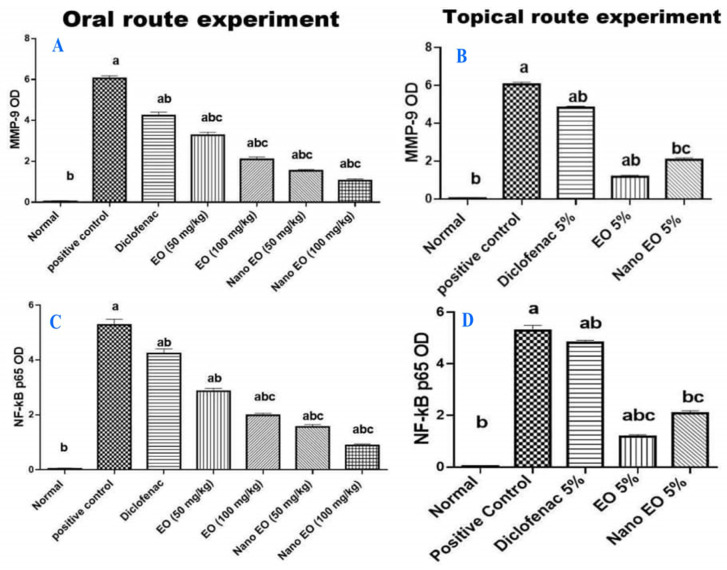
The quantitative image analysis of both MMP-9 and NF-κB p65 expression in in the oral (**A**,**C**) and topical * (**B**,**D**) experiments presented as OD of the positive brown color in five microscopic fields using image analysis software (Image J, 1.46a, NIH, USA). a—Significantly different from normal group at *p* < 0.05. b—Significantly different from positive control group at *p* < 0.05. c—Significantly different from diclofenac group at *p* < 0.05.

On the other hand, the topical application of soyabean showed non-significant decrease in both markers expression (Figure 7h and Figure 8h), whilst the topical treatment with the ABSEO (5%) (Figure 7i and Figure 8i) had a more curative effect than did the topical application of the ABSEO nanoemulsion (5%) (Figure 7j and Figure 8j) and showed a more significant reduction in marker expression. The topical application of diclofenac (Figure 7k and Figure 8k) showed no curative effect and did not reveal any significant decrement in both markers’ expression. Figure 9 represents the quantitative image analysis of the immune expression of each marker expressed as optical density (OD) in five different microscopic fields.

## 4. Discussion

The hydrodistillation of the fresh shoots of *A. bidiwillii* cultivated in Egypt afforded dark yellow oil with a pungent odor. The earlier reports described that the different *Araucaria* species afforded high EOs yields [1,20], and the shoots of *A. bidiwillii* yielded ≈0.47% (*v/w*) of EO. Herein, the extracted ABSEO was obtained with a greater yield than the other plant parts, such as Egyptian foliage (0.05%; *v/w*) [1] and Australian leaf (0.04%; *v/w*) [19]. These variations might be ascribed on the fact of the strong correlation of the yield and chemical compositions of the ABSEOs with the used plant part and variety [39], collection places, environmental conditions, and others [40].

From all identified compounds, the terpenoids were found as the main characteristic for all the chemical components, including hydrocarbon and alcoholic forms of mono-, sesqui-, and di-terpenoids. The abundance of the terpenes in the present work is completely agreed with the published data from *Araucaria* plants in general [20], and *A. bidiwillii* especially [1,19]. 

Monoterpenes represented the main components with a relative concentration of 42.94%, mainly including monoterpene hydrocarbons (40.04%) in addition to traces of monoterpene alcohols (2.90%). This result is not compatible with the described data of EOs of Egyptian foliage and Australian leaf of the same plant in which the monoterpenes are traces, 1.72% [1] and 0.60% [19], respectively. Twelve monoterpene hydrocarbons were identified, consisting of α-pinene (16.21%), D-limonene (14.22%), and sabinene (4.12%). These three major monoterpene hydrocarbons were found as traces and/or completely absent from ABSEOs of Egyptian foliage [1] and Australian leaf of *A. bidiwillii* [19]. However, preponderance of these compounds was reported in EOs of numerous *Araucaria* species, such as the Egyptian resin [20], Australian leaf of *A. heterophylla* [19], Indian fresh foliage of *A. cunninghamii* [21], Australian leaf of *A. hunsteinii*, and Australian leaf of *A. montana* [19], while only five monoterpene alcohols were assigned, involving *δ*-terpineol (2.21%) as major compound. The scarcity of the oxygenated forms of monoterpene in EOs are already usual in the early described data of EOs of Egyptian foliage [1] and Australian leaf of *A. bidiwillii* [19] as well as the other *Araucaria* eco-plants [20,21].

The sesquiterpenes were found to be the second main constituents, with a relative concentration of 31.66%, including sesquiterpene hydrocarbons (18.82%) and alcohols (12.84%). The abundance of sesquiterpenes in EOs in the present work is already harmonious with those of the Egyptian foliage of *A. bidiwillii* [1] but is in contrast with Australian leaf [19]. The phenomena of the abundance of sesquiterpenes in EOs of *Araucaria* species are non-common, with exception of the Egyptian and Indian resin of *A. heterophylla* [20,21], and Indian resin of *A. cunninghamii* [21]. Twelve sesquiterpene hydrocarbons were characterized, in which germacrene D (6.69%), β-humulene (4.14%), and bicyclogermacrene (2.31%) are the major components. Germacrene D was reported from EO of Egyptian foliage of *A. bidiwillii* [1] with absence of the other two compounds. In the other side, eleven sesquiterpene alcohols were identified with a total relative concentration of 12.84%, from which *tau*-muurolol (3.49%) and α-cadinol (3.46%) are the major components that were reported before in the ABSEO of the Egyptian foliage of *A. bidiwillii* [1]. Additionally, [41] described the presence of germacrene D with major concentration in EO of the leaves *A. bidiwillii*. 

Generally, EOs derived from the plant resources consisted of complex mixtures of mainly mono- and sesqui-terpenes, carotenoid and apocarotenoid derived compounds, and other hydrocarbons with traces of diterpenoids [42]. Based upon the previous data, the plants belonging to the *Araucaria* genus represented a remarkable exception via the ability of these plants to elaborate biosynthetically high concentration of volatile diterpenes [1,19,20,21]. The data presented in this work deduced this exception by the ability of *A. bidiwillii* to build enzymatically consistent diterpenes with a relative concentration of 23.74%. Three compounds were categorized as the total characterized diterpene including two diterpene hydrocarbons, beyerene (20.81%) and kaur-15-ene (1.07%), along with one diterpene alcohol, manool (1.86%). In 2000, Pietsch and Konig documented the abundance of the beyerene and the 15- and/or 16- kaurenes in *Araucaria* species especially the leaves of *A. bidiwillii* [41]. The existence of diterpenes with high concentration was totally agreed with the published data of Egyptian foliage [1] and Australian leaf [19]. The prevalence of the diterpene hydrocarbon compound, beyerene, was already reported in EO of foliage of Egyptian *A. bidiwillii* [1], while it was totally un-described in Australian leaf [19].

The results of droplet size diameter of the prepared nanoemulsion revealed that the mean droplet size within critical nano size was <100 nm. The concentration of Tween 80 and the time spent sonicating were also critical factors in the creation of nanoemulsions [26]. The mean droplet size diameter of ABSEO nanoemulsion was 106 nm, with 25% of the distribution measuring less than 62.3 nm, and 75% measuring less than 153.5 nm [20]. When the surfactant couple’s hydrophile–lipophile balance (HLB) value correlates with the oil’s HLB value, a small droplet size of nanoemulsion may be created [27]. From the zeta potential value, the surface potential of nanoemulsion droplets is linked to the zeta value. The zeta potential value was shown to be connected to the stability of nanoemulsion formulation. When the zeta potential is greater than 30 mV, the nanoemulsion is at its most stable [43]. 

Our findings revealed that the inflammatory mediators IL-1β and IL-8 were elevated as a result of decreased carrageenan-induced inflammation, which signified the inhibitory effect of both ABSEO and ABSEO nanoemulsion in both oral and topical treatment when compared to control groups. Our study showed that effective anti-inflammatory properties of ABSEO and its nanoemulsion administered to rats either orally or topically was evidenced by substantial abolishing NO and PGE release, as previously mentioned by [44]. In the current study, we evaluated the underlying mechanism exerted by the ABSEO and its nanoemulsion as an anti-inflammatory treatment in association with the NF-κβ pathway by evaluating the immune expression of NF-κβ p65 protein marker showing variable degrees of decreased expression via oral administration or topical application. Oral administration and topical application of ABSEO and its nanoemulsion showed a significant decrease in immune expression of MMP-9 in paw tissues. Our results agreed with those of [45]. From all the previous studies, we can conclude that the anti-inflammatory effect of ABSEO and its nanoemulsion might be associated with the inhibitory effect on the release of proinflammatory and inflammatory biomarkers.

In the same route of cytokines mediators, yeast-induced pyrexia is a widely used animal model for fever. The incidence of pyrexia involves many mediators, such as interleukins IL-6, IL-1β, IL-8, and TNF-α, as recorded in previous studies [46]. Moreover, PGE2 generated under the action of Cox-2 is an important mediator, which induces pyrexia [47]. Our results showed that oral administration of ABSEO and its nanoemulsion in both dose levels significantly reduced fever in pyrexia-induced rats 24 h after treatment. The anti-inflammatory effect of the ABSEO and its nanoemulsion evidenced by inhibition of IL-1β, IL-8, and PGE2 expression might be the underlying mechanism for the antipyretic effect of the drugs as these drugs were able to suppress the inflammatory pathway generating pyrogenic cytokines.

The present results exhibited that the used concentrations of ABSEO and its nanoemulsion cause the significant inflammations inhibitory effects on carrageenan-induced rats that deduced by the biochemical and pathological as well as immunohistochemical assays compared to control and the reference drug groups. Additionally, the ABSEO and its nanoemulsion showed strong antipyretic potentialities. It is a scientific fact that there is a fundamental relationship between the biological potentialities of the plant extracts and their chemical components [48]. The live bodies in in vivo evaluations were deduced to have significant capabilities of absorption of the EOs derived from plants in addition to the attaching to the digestive tract lining [49]. Some documents reported that about 98% of olive oil was absorbed in in vivo studies in humans with a dose–response manner [49,50]. This fact supported the significant activity of the ABSEO as anti-inflammatory and antipyretic potentialities. 

Based on this fact, the significant anti-inflammatory and antipyretic effects of ABSEO and its nanoemulsion could be ascribed to the chemical components of ABSEO, predominately the main constituents. The chemical profiling of ABSEO revealed the prevalence of terpenoids, including mono-, sesqui- and diterpenes. Monoterpenes with a relative concentration of 42.94% represented the main bioactive mediators. Anti-inflammatory and antipyretic active plants ABSEOs were chemically found enriched with terpenoids, especially mono and sesquiterpenes [20,51,52,53,54]. α-Pinene was described as a critical component in ABSEOs with potent inhibitory action against paw edema and inflammations via cytokines secretion [55,56]. According to [57,58], α-pinene can act as an anti-inflammatory via the suppression of IL-1β, NF-κB, and iNOS and nuclear translocation of NF-kappa B. The documented in vivo results of α-pinene revealed the fact of the significant role of this compound as an anti-inflammatory agent even at high and/or low concentrations [59]. Several documents deduced this role of α-pinene as a widely distributed compound in ABSEOs. The reported data showed that the EOs derived from two plants, *Pinus* and *Abies*, have potent anti-inflammatory effects because of the abundance of α-pinene [55,56]. Additionally, [20] stated that the EO of *A. heterophylla* has a significant anti-inflammatory due to ≈45% of α-pinene that inhibits the inflammation mediators, TNF-α, IL-1β, and IL-6. 

Additionally, some reported data deduced the significant role of caryophyllene, limonene, and sabinene as anti-inflammations agents in both in vitro and in vivo models, including carrageenan-induced rat paw [60]. Limonene and its derivatives were documented to play main roles in the reduction of inflammatory reaction by inhibition of the activities 5-lipoxygenase, neutrophils [55]. EOs of some plants, such as *citrus* species [61], *A. heterophylla* [20], and *Ageratum fastigiatum* [55,62], were found to have potent anti-inflammatory potentialities due to the high content of sesquiterpene compounds, such as germacrene-D and β-humulene. The earlier reported data exhibited that the ABSEO of foliage of *A. bidiwillii*, enriched with beyerene (35.65%), has anticancer activity against the human cancer cells via the caspase-dependent apoptosis [1]. The authors of [63] stated that the activation of caspase caused two pathways: i) amending of the dysfunctional cells death and ii) decreasing the inflammation via secretion of cytokines in several inflammation diseases. Based upon these two reported datasets, beyerene, as the main compound in our study, might act as an activator of caspase and therefore cause inhibition of the inflammation mediators along with apoptosis. Behind the main constituents, the other terpenoids, including mono-, sesqui-, and diterpenes, might be significant inhibitory agents, singularly and/or synergistically, of inflammations according to the previous in vivo and in vitro studies including carrageenan-induced rat paw [60].

Bilia et al., 2014 [64] described that the nanoemulsion of the EOs is more stable and increases its cellular absorption. This fact was confirmed in [20], which found that the nanoemulsion of *A. hereophylla* is more active than the EO itself as anti-inflammatory and antipyretic along with increasing of the activity in a dose-dependent manner. With complete harmony of these documented studies, our results exhibited that the ABSEO nanoemulsion has significant oral anti-inflammatory and antipyretic activities, and this activity increased in a dose-dependent manner. This activity might be attributed to direct oral administration of ABSEO nanoemulsion, which increases the absorption of the cells of the nanoemulsion, along with its stability and the decrease in its volatility. 

## 5. Conclusions

The chemical analysis of the derived EO from the shoots of the *A. bidiwillii* cultivated in Egypt revealed the identification of 43 terpenoid components. Beyerene, α-pinene, d-limonene, and germacrene D were identified as the main compounds. A nanoemulsion form of ABSEO was prepared. The current results deduced that the ABSEO and its nanoemulsion have significant in vivo oral and topical anti-inflammatory along with antipyretic potentialities by suppression of proinflammatory cytokines as well as downregulation of PGE2. Likewise, the histopathological studies showed a marked regeneration of paw tissues. Accordingly, we can recommend the use of ABSEO and its nanoemulsion as promising anti-inflammatory and antipyretic agents.

## Figures and Tables

**Table 1:** Essential oil components of the shoots of *Araucaria bidiwillii*.

No	Current Study					% of Combined Compounds in Previous Studies	
	Rt ^a^	Rel. Conc. % ^b^	Component Name ^c^	M.F. ^d^	KI ^e^	Egyptian Foliage ^f^	Australian Leaf ^g^
Monoterpene Hydrocarbons							
1	3.72	0.85 ± 0.02	Tricyclene	C_10_H_16_	921	---	---
2	3.92	16.21 ± 0.78	α-Pinene	C_10_H_16_	932	1.52	0.5
3	4.28	0.38 ± 0.02	Camphene	C_10_H_16_	946	---	---
4	4.74	4.12 ± 0.11	Sabinene	C_10_H_16_	969	---	---
5	4.87	1.51 ± 0.05	β-Pinene	C_10_H_16_	979	0.02	traces
6	5.11	0.41 ± 0.01	α-Myrcene	C_10_H_16_	990	0.03	traces
7	5.49	0.16 ± 0.01	α-Phellandrene	C_10_H_16_	1002	---	---
8	5.56	0.25 ± 0.02	Pseudolimonene	C_10_H_16_	1004	---	---
9	5.81	0.69 ± 0.04	α-Terpinene	C_10_H_16_	1014	---	---
10	6.13	14.22 ± 0.16	D-Limonene	C_10_H_16_	1024	0.11	0.1
11	6.94	1.03 ± 0.04	γ-Terpinene	C_10_H_16_	1054	0.02	---
12	7.72	0.21 ± 0.02	α-Terpinolene	C_10_H_16_	1086	---	---
Monoterpene Alcohols							
13	9.52	0.17 ± 0.02	*trans*-Pinocarveol	C_10_H_16_O	1139	---	---
14	10.54	0.11 ± 0.01	Borneol	C_10_H_18_O	1165	---	---
15	10.78	2.21 ± 0.06	δ-Terpineol	C_10_H_18_O	1166	---	---
16	11.39	0.32 ± 0.03	4-Terpineol	C_10_H_18_O	1174	---	---
17	16.12	0.09 ± 0.01	Terpinen-4-yl acetate	C_12_H_20_O_2_	1299	---	---
Sesquiterpene Hydrocarbons							
18	16.8	0.71 ± 0.04	α-Copaene	C_15_H_24_	1375	0.20	traces
19	17.27	0.13 ± 0.01	β-Elemene	C_15_H_24_	1389	0.20	---
20	18.2	2.16 ± 0.10	β-Caryophyllene	C_15_H_24_	1408	0.19	0.1
21	19.36	4.14 ± 0.13	β-Humulene	C_15_H_24_	1436	---	0.1
22	19.98	0.43 ± 0.02	γ-Muurolene	C_15_H_24_	1478	0.79	---
23	20.17	6.69 ± 0.21	Germacrene D	C_15_H_24_	1484	5.53	0.2
24	20.44	0.10 ± 0.01	β-Selinene	C_15_H_24_	1489	---	---
25	20.6	2.31 ± 0.08	Bicyclogermacrene	C_15_H_24_	1500	---	0.2
26	20.74	0.22 ± 0.01	α-Muurolene	C_15_H_24_	1500	---	---
27	21.19	0.40 ± 0.02	δ-Amorphene	C_15_H_24_	1512	---	---
28	21.3	1.43 ± 0.04	γ-Cadinene	C_15_H_24_	1513	2.03	0.1
29	22.15	0.10 ± 0.01	α-Calacorene	C_15_H_20_	1544	---	---
Sesquiterpene Alcohols							
30	22.71	1.01 ± 0.02	trans-Nerolidol	C_15_H_26_O	1531	13.66	---
31	22.92	0.07 ± 0.01	Spathulenol	C_15_H_26_O	1577	1.09	3.2
32	23.17	0.20 ± 0.02	Globulol	C_15_H_24_O	1590	---	0.2
33	23.66	0.64 ± 0.03	Viridiflorol	C_15_H_26_O	1600	---	---
34	23.95	0.56 ± 0.05	Ledol	C_15_H_26_O	1602	---	---
35	24.47	0.13 ± 0.01	Fonenol	C_15_H_26_O	1627	---	---
36	24.62	1.17 ± 0.07	T-Cadinol	C_15_H_26_O	1640	1.76	---
37	25.1	3.49 ± 0.09	T-Muurolol	C_15_H_26_O	1642	2.51	---
38	25.16	2.03 ± 0.08	Cubenol	C_15_H_26_O	1645	---	---
39	25.49	3.46 ± 0.12	α-Cadinol	C_15_H_26_O	1652	---	---
40	25.82	0.08 ± 0.01	Khusinol	C_15_H_24_O	1823	---	---
Diterpene Hydrocarbons							
41	32.7	20.81 ± 0.21	Beyerene	C_20_H_32_	1931	35.65	---
42	33.41	1.07 ± 0.06	Kaur-15-ene	C_20_H_32_	1997	1.37	---
Diterpene Alcohols							
43	34.22	1.86 ± 0.07	Manool	C_20_H_34_O	2056	---	---
		40.04	Monoterpene hydrocarbons				
		2.90	Monoterpene alcohols				
		18.82	Sesquiterpene hydrocarbons				
		12.84	Sesquiterpene alcohols				
		21.88	Diterpene hydrocarbons				
		1.86	Diterpene alcohols				
		98.34	Total				

^a^ Retention times, ^b^ Rel. Conc.%: Relative concentration, ^c^ EO constituents identification was constructed via compound mass (MS) spectra and Kovats’ retention indices (KI) with those of Wiley spectral library collection and NIST library databases, ^d^ MF: Molecular formula, ^e^ Kovats’ retention index according to literature on a DB-5 column in reference to *n*-alkanes, ^g,f^ percent of combined compounds in EO of Egyptian foliage ([1]), and Australian leaf ([19] of *Araucaria bidiwillii*).

**Table 2:** Anti-inflammatory effect of oral administration of ABSEO and its nanoemulsion (50 and 100 mg/kg) against carrageenan-induced inflammation with referring to topical diclofenac.

Groups	Baseline	1 h		2 h		3 h		4 h	
	Paw Thickness (mm)	% Edema	% Inhibition	% Edema	% Inhibition	% Edema	% Inhibition	% Edema	% Inhibition
Positive control	2.39 ± 0.15	53.9 ± 9.37	-------	72.2 ± 3.54	-------	83.0 ± 5.25	-------	76.8 ± 6.36	-------
Diclofenac (30 mg/kg)	2.14 ± 0.1	46.7 ± 6.60	13.36	27.4 ± 7.7	62.04	18.5 ± 3.2	77.7	9.7 ± 1.3	76.7
EO (50 mg/kg)	2.14 ± 0.01	72.5 ± 10.7	-------	73.8 ± 9.4	-------	74.5 ± 8.4	10.24	69.2±14.3	9.89
EO (100 mg/kg)	2.27 ± 0.08	40.4 ± 7.3	25.04	82.6 ± 15.3	-------	76.4 ± 18.2	7.95	73.5 ± 17.9	4.29
Nano EO (50 mg/kg)	2.29 ± 0.07	44.3 ± 8.2	17.8	68.6 ± 11.25	4.99	57.2 ± 13.6	31.08	40.3 ± 4.45	47.5
Nano EO (100 mg/kg)	2.39 ± 0.03	40.1 ± 8.7	25.6	62.6 ± 8.9	8.75	42.7 ± 6.9	48.55	32.1 ± 6.2	58.2

Paw thickness of baseline is expressed as (mean ± SE). % of Edema rate is represented as (mean% ± S.E). The percentage of inhibition was calculated as: Ec − Et/Ec. Ec = edema rate of control group; Et = edema rate of treated group. Statistical analysis was carried out by one-way analysis of variance (ANOVA) followed by Tukey–Kramer test for multiple comparisons. Carr. = carrageenan; Diclo. = Diclofenac.

**Table 3:** Topical anti-inflammatory effects of ABSEO and its nanoemulsion (5% in soyabean oil) against carrageenan-induced inflammation with refer to diclofenac.

Groups	Baseline	1 h		2 h		3 h		4 h	
	Paw Thickness (mm)	% Edema	% Inhibition	% Edema	% Inhibition	% Edema	% Inhibition	% Edema	% Inhibition
Positive control	2.39 ± 0.15	53.9 ± 9.37	-------	72.2 ± 3.54	-------	83.0 ± 5.25	-------	76.8 ± 6.36	-------
Diclofenac 5%	2.15 ± 0.13	39.3 ± 8.86	27.09	95.3 ± 17.3	-------	124.6 ± 19.8	-------	95.8 ± 16.68	-------
EO 5%	2.3 ± 0.2	36.4 ± 5.75	32.47	43 ± 19.64	40.44	32.6 ± 19.77	57.55	33.3 ± 11.49	59.87
Nano EO 5%	2.28 ± 0.07	26.4 ± 8.16	51.02	45.2 ± 8.64	37.39	67.2 ± 6.8	19.04	57.8 ± 1.01	24.7

Paw thickness of baseline is expressed as (mean ± SE). % of Edema rate is represented as (mean% ± S.E). The percentage of inhibition was calculated as: Ec − Et/Ec. Ec = edema rate of control group; Et = edema rate of treated group. Statistical analysis was carried out by one-way analysis of variance (ANOVA) followed by Tukey–Kramer test for multiple comparisons. Carr. = carrageenan; Diclo. = Diclofenac.

## Data Availability

Data are contained within the article.

## Sample Availability

Samples of the compounds are not available from the authors.
